# Macrochirality of Self-Assembled and Co-assembled Supramolecular Structures of a Pair of Enantiomeric Peptides

**DOI:** 10.3389/fmolb.2021.700964

**Published:** 2021-06-23

**Authors:** Zhen Guo, Yongshun Song, Yujiao Wang, Tingyuan Tan, Yuwen Ji, Guangxu Zhang, Jun Hu, Yi Zhang

**Affiliations:** ^1^ Key Laboratory of Interfacial Physics and Technology, Shanghai Institute of Applied Physics, Chinese Academy of Sciences, Shanghai, China; ^2^ University of Chinese Academy of Sciences, Beijing, China; ^3^ School of Science, East China University of Science and Technology, Shanghai, China; ^4^ Zhangjiang Lab, Shanghai Advanced Research Institute, Chinese Academy of Sciences, Shanghai, China

**Keywords:** amyloid peptide, chirality, self-assembly, co-assembly, macrochirality

## Abstract

Although macrochirality of peptides’ supramolecular structures has been found to play important roles in biological activities, how macrochirality is determined by the molecular chirality of the constituted amino acids is still unclear. Here, two chiral peptides, Ac-^L^K^L^H^L^H^L^Q^L^K^L^L^L^V^L^F^L^F^L^A^L^K-NH_2_ (KK-11) and Ac-^D^K^D^H^D^H^D^Q^D^K^D^L ^D^V^D^F^D^F^D^A^D^K-NH_2_ (KKd-11), which were composed entirely of either L- or D-amino acids, were designed for studying the chiral characteristics of the supramolecular microstructures. It was found that monocomponent KK-11 or KKd-11 self-assembled into right- or left-handed helical nanofibrils, respectively. However, when they co-assembled with concentration ratios varied from 1:9 to 9:1, achiral nanowire-like structures were formed. Both circular dichroism and Fourier transform infrared spectra indicated that the secondary structures changed when the peptides co-assembled. MD simulations indicated that KK-11 or KKd-11 exhibited a strong propensity to self-assemble into right-handed or left-handed nanofibrils, respectively. However, when KK-11 and KKd-11 were both presented in a solution, they had a higher probability to co-assemble instead of self-sort. MD simulations indicated that, in their mixtures, they formed nanowires without handedness feature, a good agreement with experimental observation. Our results shed light on the molecular mechanisms of the macrochirality of peptide supramolecular microstructures.

## Introduction

Chirality is a universal phenomenon in biological systems (Hegstrom and Kondepudi, 1990; Goldanskii and Kuzmin, 1991; Bada, 1995; Liu et al., 2015). It has been a consensus that the chirality of biomolecules plays important roles in biometabolic processes and biological activities (Mason, 1985; Barron, 1986; Meiring, 1987; Mason, 1988; Avetisov et al., 1991; Compton and Pagni, 2002; Valev et al., 2013; Wang et al., 2013). Molecular chirality in amino acids, peptides, or proteins is essentially critical as they are the structural and functional basis of the organism (Kondepudi and Nelson, 1985; Mason, 1986; Goldanskii and Kuzmin, 1988; Campbell, 1990; Bonner, 1991; Chelaflores, 1994; Friedman, 1999). In recent years, in addition to the chirality at the molecular level, the effects of the chirality of the supramolecular structures of proteins and peptides are attracting increasing attention due to discoveries of a series of extraordinary phenomenon (Gutman et al., 1988; Surewicz and Mantsch, 1988; Fassihi, 1993; Boyd et al., 2011; Caglioti et al., 2011; Taniguchi et al., 2011; Gleiser and Walker, 2012; Ariga et al., 2020). For example, it has been found that the chirality of peptide nanofibers in hydrogel could affect cell adhesion and proliferation (Das et al., 2013; Liu et al., 2014).

Understanding the chirality of the peptide supramolecular structure is an important issue (Quack, 2002; Deamer et al., 2007; McKellar, 2009; Wang et al., 2013). Many isomeric peptides with the same amino acid composition have shown different self-assembly behaviors, which resulted in different supramolecular morphologies (Dzwolak et al., 2007; Rubin et al., 2008; Adhikari et al., 2011; Wang et al., 2017). A lot of works have been done on revealing the relationship between the chirality of the amino acids in peptide sequences and the macrochirality of the peptide’s supramolecular structures. It is generally accepted that amino acid conformations control the morphological and chiral features of the self-assembled peptide nanostructures (Zhou et al., 2019). Some studies reported that L-peptides (peptides comprised of L-amino acids) self-assembled into left-handed nanostructures, while the D-peptides (peptides comprised of D-amino acids) formed right-handed nanostructures (Rubin et al., 2008; Adhikari et al., 2011). It was also found that the homochiral tripeptides (^L^F^L^F^L^V and ^D^F^D^F^D^V) exhibited poor self-assembling properties; however, the heterochiral peptides (^D^F^L^F^L^V and ^L^F^D^F^D^V) were able to self-assemble into chiral nanofibrils (Marchesan et al., 2014). In another example, peptides with sequences of Ac-^X^I_3_^Y^K-NH_2_ (X = L, D, La, Da; Y = L, D) were designed and it was experimentally demonstrated that the chirality of the C-terminal lysine residue (K) determined the helical chirality of the assembled peptide nanofibrils, while the N-terminal isoleucine residue (I) determined the characteristic circular dichroism (CD) signals of the assemblies (Wang et al., 2017). It was found that a heterochiral dipeptide (^D^F^L^F) exhibited a high propensity to form fibrillar structures independently of the solvent composition, whereas the morphologies of the assemblies of homochiral dipeptide (^L^F^L^F) changed with the environment (Gil et al., 2020). Another study reported that equimolar mixtures of enantiomeric amphipathic peptides (L- and D-(FKFE)2) did not self-sort but rather co-assemble into fibrils that contained alternating L- and D-peptides in a rippled *ß*-sheet orientation (Swanekamp et al., 2012a). Although there are plenty of literature (Cornelissen et al., 2001; Dzwolak et al., 2007; Adhikari et al., 2011; Volpatti et al., 2013; Zhao et al., 2013; Zhou et al., 2016; Wang et al., 2017; Yue and Zhu, 2019; Zhou et al., 2019) reporting the factors that influence the macrochirality of self-assembled peptide supramolecular structures, currently it is still not thoroughly understood how the macrochirality is determined. Therefore, investigation on hierarchical self-assembling peptides comprising of chiral amino acids would shed light on the formation mechanism of macrochirality.

In this paper, the characteristics of the supramolecular microstructures of two chiral peptides, Ac-^L^K^L^H^L^H^L^Q^L^K^L^L^L^V^L^F^L^F^L^A^L^K-NH_2_ (KK-11) and Ac-^D^K^D^H^D^H^D^Q^D^K^D^L ^D^V^D^F^D^F^D^A^D^K-NH_2_ (KKd-11), were studied. The KKd-11 and KK-11 were previously developed in our group for long-term inhibiting the formation of biofilms (Guo et al., 2021). Here, it was found that the microscopic morphology of the assembled peptide structure was highly related to the intrinsic chirality of the peptides, in which KK-11 self-assembled into right-handed helical nanofibrils, whereas KKd-11 self-assembled into left-handed helical nanofibrils. However, when the two peptides were mixed to co-assemble in molecular ratios ranging from 1:9 to 9:1, only achiral nanowire-like structures were observed. Through theoretical simulations, it was found that the hybrid interacting structures of KK-11andKKd-11 were more stable than the structures formed by the monocomponent peptides. Besides, simulations also revealed that the self-assembled structures of monocomponent peptides exhibited a strong propensity to twist. In contrast, the co-assembled structures exhibited no handedness propensity, a good agreement with experimental observations. Our studies shed light on the molecular mechanisms of the macrochirality of peptide supramolecular microstructures.

## Materials and Methods

### Materials

The peptides KK-11 and KKd-11 were purchased from China Peptides Co., Ltd. The peptides had a purity of 97% which was verified by high-performance liquid chromatography (HPLC) and mass spectrum (MS). The peptide powder was store at −20°C before use. Phosphate Buffer Saline (PBS), hydrochloric acid (HCl), sodium hydroxide (NaOH), and medium were purchased from Sinopharm Chemical Reagent (China). All aqueous solutions were prepared with deionized (DI) water from Milli-Q-Water (Millipore Corp, 18.2 MΩ/cm at 25°C).

### Peptide Co-assembly

KK-11 and KKd-11 powers were mixed and dissolved in PBS for co-assembly under varied peptide concentration ratios. The total peptide concentration was set at 5 mg/ml unless otherwise stated.

### Atomic Force Microscopy

A commercial AFM instrument (Nanoscope VIII, Bruker) equipped with a 100-μm scanner was used to measure the morphology of the assembled peptide structures. A silicon nitride cantilever (XSC-11, MIKROMASCH) with a nominal force constant of 7 N/m was used. Experiments were carried out in the air in Peak Force Quantitative Nano Mechanical AFM (PF-QNM) mode. Newly cleaved mica was used as a substrate. All images were analyzed using the Nanoscope Software (Nanoscope Analysis Version 1.40) supplied by the AFM manufacturer.

### Transmission Electron Microscopy

TEM images were taken by a Tecnai G2 F20S-TWIN microscope under a high vacuum. To prepare the sample, a drop of peptide solution (5 μL) was first placed on a carbon-coated copper grid and adsorbed for 1 min. Then the remained sample residual was removed by a filter paper. The loaded grid was washed with double distilled water, then stained with 2% (w/v) uranyl acetate.

### Scanning Electron Microscopy Characterization

Silicon wafer was used as the substrate for sample preparation. A gold film was sprayed on the silicon wafer after the sample was deposited on it. SEM instrument model: Carl Zeiss AG, LEO 1530VP.

### Circular Dichroism Spectroscopy

CD spectra were recorded over the wavelength range of 190–260 nm by using a Chirascan (Applied Photophysics, United Kingdom) with a 0.1 cm path length sample cell. All CD measurements were performed at room temperature using a bandwidth of 1.0 nm, a step interval of 1 nm, and a scanning speed of 50 nm min^−1^. Each CD spectrum was averaged from three scans, and the corresponding baseline of buffer was subtracted from the sample spectrum. The secondary structures of peptide samples were analyzed using the CDNN 2.1 program.

### Fourier Transform Infra-Red Spectroscopy

The peptide solution was processed and placed on clean BaF_2_ glass. The FTIR spectra were recorded using a Nicolet 6700 FTIR spectrometer with a Continuum XL FTIR microscope (Thermo Fisher Scientific, United States) at a spectral resolution of 4 cm^−1^. Infrared spectra were recorded between 1800 and 1200cm^−1^. All resulting spectra were corrected for the blank background around sample absorption. Spectra were processed using the OMNIC 9.2 (Thermo Fisher Scientific, United States) for smoothing and normalization.

### Thioflavin T Fluorescence Measurement

The assembly kinetics of KK-11 and KKd-11 peptide were monitored using a dye ThT, the fluorescence of which was dependent on the formation of amyloid aggregates/fibrils. ThT fluorescence measurement was performed at 37°C using a Thermo Scientific Fluoroskan Ascent (Thermo Fisher Scientific, United States) in quiescence. The excitation and emission wavelengths were 440 and 484 nm, respectively. Fluorescence was measured immediately after the mixture was made with the reaction mixture containing 10 µM ThT. At least three different samples were independently measured to get an average fluorescence intensity.

### Molecular Dynamics Simulation Methods

In this work, a system of two KK-11 and two KKd-11 peptides starting from extended conformations solvated in water was first constructed. TIP4P explicit water model (Jorgensen et al., 1983) was used to solvate the peptides. Five independent 1-μs instances were simulated, with the numbers of water molecules range from 6,775 to 6,794. Besides, 12 chloride ions were added to neutralize the systems. The ion strength in such a system was close to that in the 0.01 M PBS buffer that we used in experiments. The simulations were conducted in a constant NPT ensemble at a pressure *p* = 1 atm and a temperature T = 300 K. The simulation box was initially set as 6 nm × 6 nm × 6 nm. During the simulation, they stayed almost constant with the volume averages range from 213.3 to 214.5 nm^3^ and a root mean square deviation (RMSD) of 0.7 for all instances. The system temperature was kept constant by a Nosé-Hoover thermostat (Hoover, 1985) with a coupling time of 0.1 ps, and the system pressure was kept constant by a Parrinello-Rahman barostat (Parrinello and Rahman, 1981) with a coupling time of 0.5 ps The electrostatic interactions were treated with the particle-mesh-Ewald method (Darden et al., 1993; Essmann et al., 1995), and both the cut-off of the van der Waals (VDW) interactions and the cut-off of the electrostatic interactions in real space were set to be 1.2 nm.

Three assembled *ß*-strands systems were then constructed. Corresponding to the experimental part, these were the KK-11, KKd-11, and mixed KK-11/KKd-11 systems. For each system, the box was set as 25 nm × 25 nm × 100 nm and was kept constant during the simulations. A total of 162 peptides were used to construct the initial structures. Solvent effects were taken into account by the GBSA implicit-solvent model (Onufriev et al., 2004). The system temperature was initially set to 10 K and then increased to 203 K. After 500 ps equilibration, the temperature was raised to 293 K and kept constant for at least 1 ns using the Berendsen thermostat with a time constant of 0.1 ps

All MD simulations were carried out by using the GROMACS 4.6.7 software package (Hess et al., 2008). Both KK-11 and KKd-11 peptides were modeled by the OPLS-AA force field (Kaminski et al., 2001). Since the force field parameters were independent of chirality, the parameters originally derived from L-amino acids were directly applied to D-amino acids.

## Results and Discussion

### KK-11 or KKd-11 Self-Assembled Into Helical Nanofibrils

**FIGURE 1:**
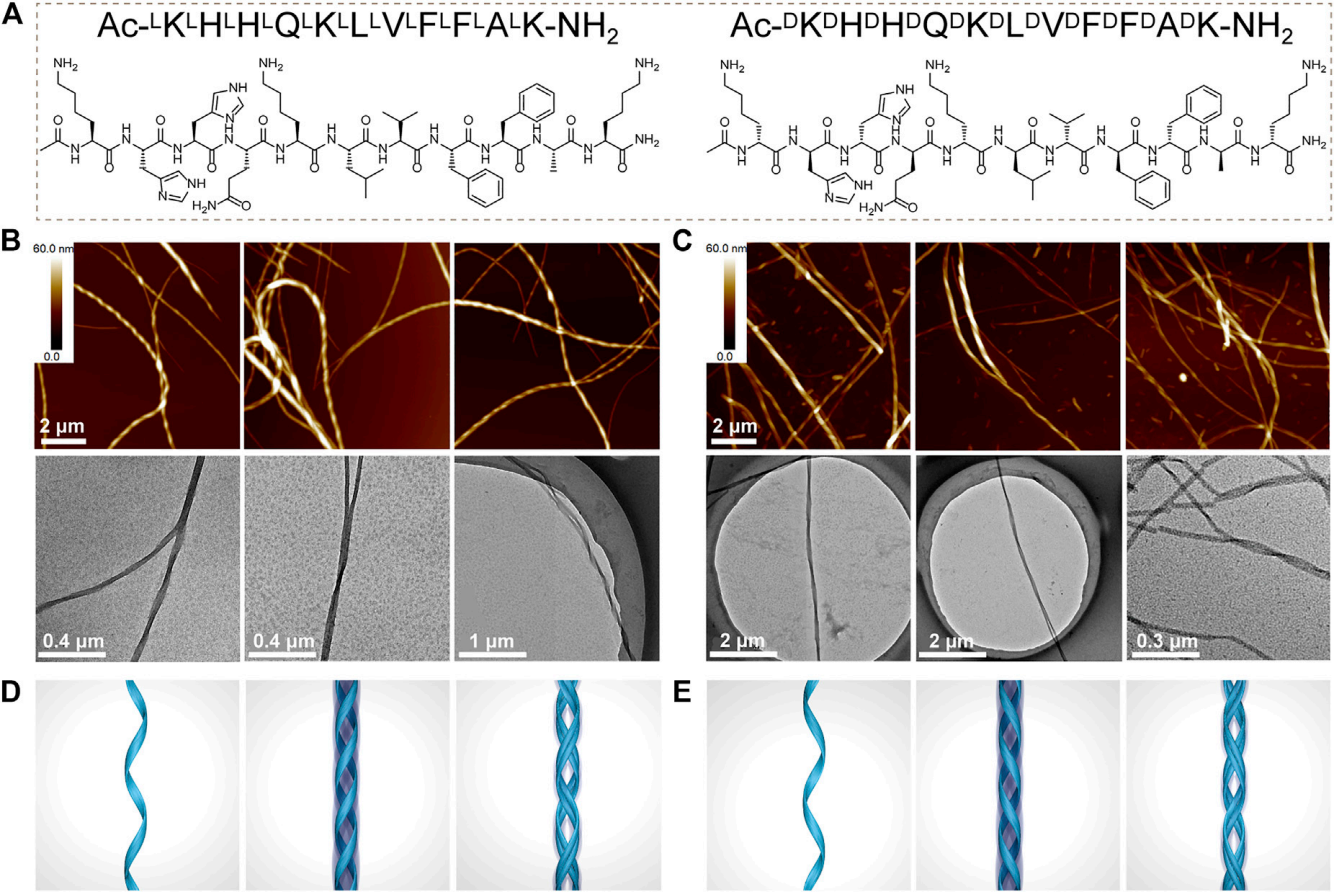
**(A)** Chemical structures of Ac-^L^K^L^H^L^H^L^Q^L^K^L^L^L^V^L^F^L^F^L^A^L^K-NH_2_ (KK-11) and Ac-^D^K^D^H^D^H^D^Q^D^K^D^L^D^V^D^F^D^F^D^A^D^K-NH_2_ (KKd-11). **(B, C)** AFM and TEM images of the assembled nanofibrils formed by KK-11 (B) and KKd-11 (C), respectively. **(D, E)** Sketch maps of helical nanofibrils formed by KK-11 (D) and KKd-11 (E).

The KK-11 or KKd-11 peptides (Figure 1A) were dissolved in PBS and then incubated with rotation at 900 rpm under 37°C for 3 days. AFM and TEM studies revealed a right-handed helical nanofibril structure formed by the KK-11 and a left-handed helical nanofibril structure by the KKd-11 (Figures 1B,C). TEM and AFM studies showed that these nanofibrils had a dimension of several micrometers in length, tens of nanometers in height, and tens of nanometers in width. Some nanofibrils tangled with each other and formed higher-level fibers. Interestingly, the helical chirality of such fibers was consistent with the fibrils that formed them, with the right-handed helix for the KK-11 fibers and the left-handed helix for the KKd-11 fibers (Figures 1B–E), respectively. Obviously, in our systems, the macrochirality of the assembled nanofibrils was determined by the chirality of the amino acids (L- or D-type) that composed the peptide (KK-11 or KKd-11).

### KK-11/KKd-11 Peptides Co-assembled Into Achiral Nanowires

**FIGURE 2:**
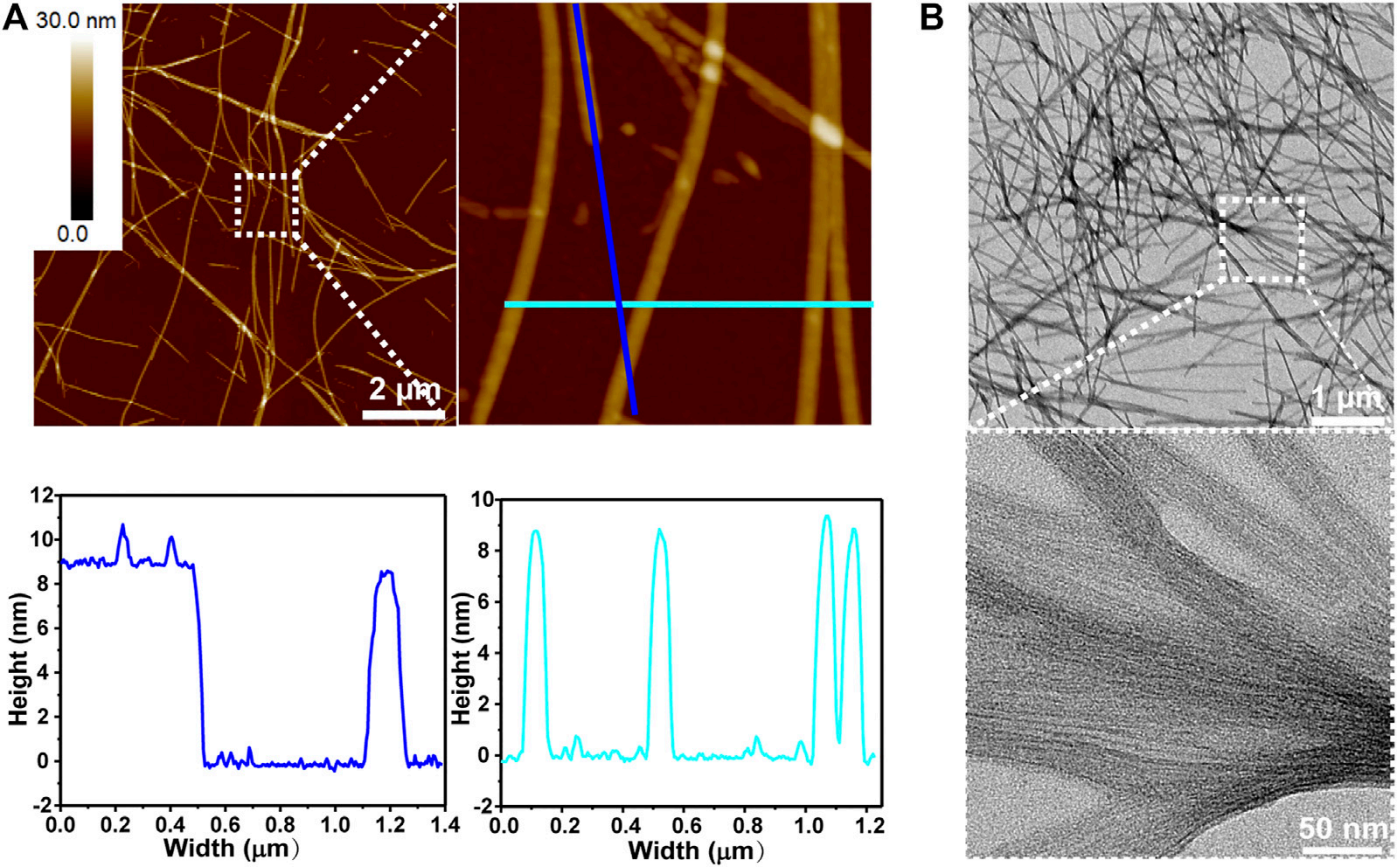
Peptide nanowire structures co-assembled by KK-11 and KKd-11 in a concentration ratio of 1:1. **(A)** AFM images **(upper)** and linear section analysis of the colored lines shown in AFM image **(lower)**. **(B)** TEM images.

When KK-11 and KKd-11 were mixed at a concentration ratio of 1: 1 and the mixture was incubated with rotation at 900 rpm under 37°C for 3 days, it was found that the two kinds of peptides co-assembled into linear nanostructures (Figure 2). However, unlike the nanofibrils formed by monocomponent peptides, both AFM and TEM revealed that the linear nanostructures were in a smooth, uniform but an achiral nanowire-like feature. AFM measurements indicated that the nanowires had a height of about 9 nm. TEM images and AFM showed that these nanowires had a dimension of several micrometers in length and tens of nanometers in width.

**FIGURE 3:**
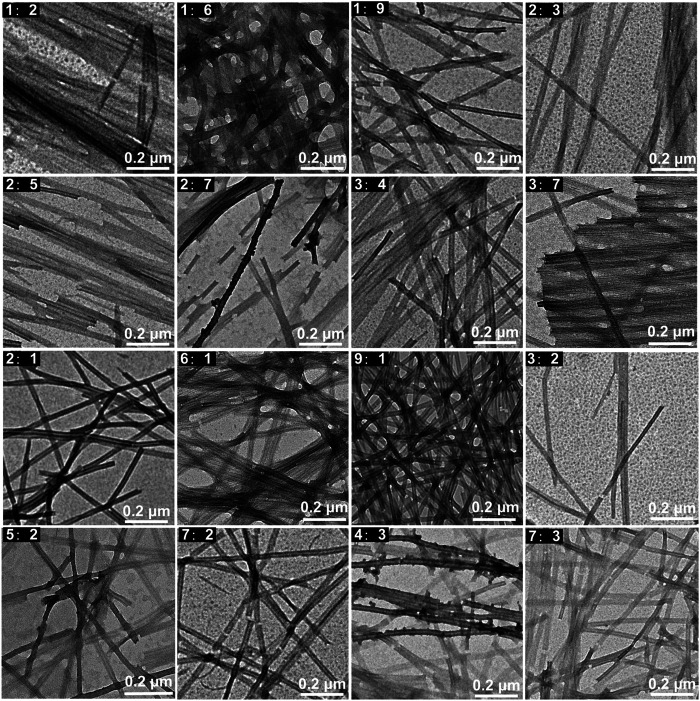
TEM images of the nanowires co-assembled by KK-11 and KKd-11 with different concentration ratios (1: 2, 1: 6, 1: 9, 2: 3, 2: 5, 2: 7, 3: 4, 3: 7, 2: 1, 6: 1, 9: 1, 3: 2, 5: 2, 7: 2, 4: 3, 7: 3).

In order to explore the effects of the concentration ratio of the two peptides on the co-assembled nanostructures, peptide mixtures in different ratios including 1: 2, 1: 6, 1: 9, 2: 3, 2: 5, 2: 7, 3: 4, 3: 7, 2: 1, 6: 1, 9: 1, 3: 2, 5: 2, 7: 2, 4: 3, 7: 3 (KK-11: KKd-11) were studied. The experimental results indicated that the two kinds of peptides co-assembled into similar linear nanowires without helix in all the studied ratios (Figure 3).

### Secondary Structures

**FIGURE 4:**
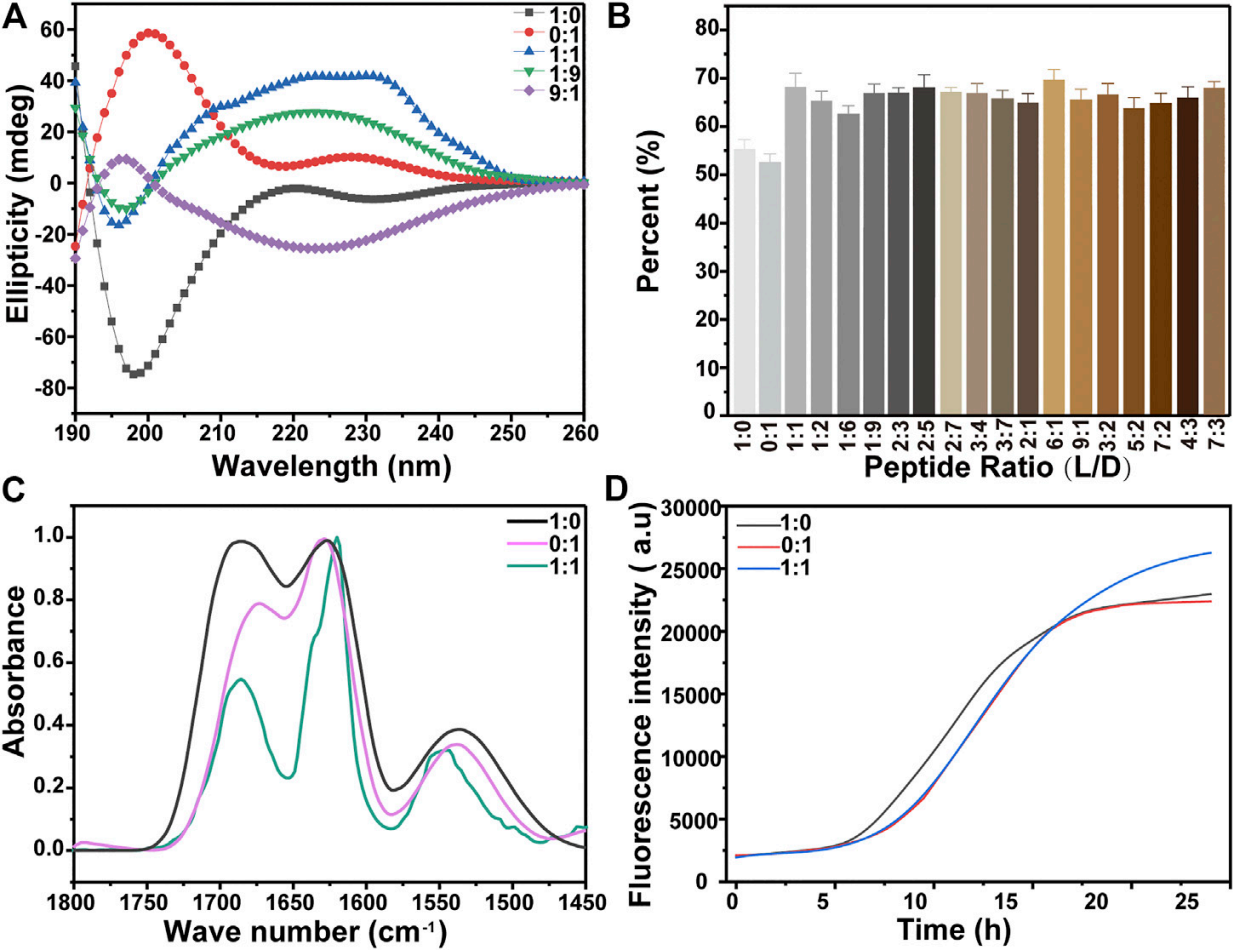
**(A)** The CD spectra of KK-11, KKd-11, and a mixture of the two peptides (1:1, 1:9, and 9:1 in ratio of KK-11:KKd-11). **(B)** Percentage of *ß*-sheet structure in the peptide, which was calculated from the CD spectra using software CDNN. (C-D) FTIR spectra **(C)** and ThT fluorescence **(D)** of the aqueous solutions of KK-11, KKd-11, and their mixture (1:1 in ration), respectively.

CD and FTIR were employed to characterize the secondary structures of the peptides assembled into nanostructures. The CD spectra of KK-11 and KKd-11 showed almost mirrored curves concerning the horizontal axis (Figure 4A), which reflect their chiral characteristics. However, the CD spectra did not show the characteristic bands of the specific secondary structures, most likely due to the superposition of the bands of multiple secondary structures. Therefore, the CD spectrum was quantitatively analyzed through a CDNN program (Greenfield, 2006). The results showed that the main secondary structures were *ß*-sheet and *a*-helix. The proportion of the two secondary structures was calculated and normalized, and the change of *ß*-sheet content was shown in Figure 4B. Infrared spectroscopy is one of the important techniques to measure the protein’s secondary structures (Surewicz and Mantsch, 1988; Dunbar et al., 2011). In our experiments, a characteristic peak at 1,628 cm^−1^was observed (Figure 4C), suggesting the existence of *ß*-sheet in the peptides.

Besides, ThT, whose fluorescence intensity was dependent on the transformation of the peptide into the *ß*-sheet structure, was used to monitor the dynamic assembly of the peptides KK-11 or KKd-11. As shown in Figure 4D, the ThT fluorescence signal had little change in the first 5 h but began to increase thereafter, indicating that the peptides were transforming to *ß*-sheet, which had a strong tendency to self-assembly. Also, from the CD spectra and ThT fluorescence intensities, it was found that the contents of *ß*-sheet in KK-11 and KKd-11 were similar. However, in the mixtures of KK-11 and KKd-11 with a wide concentration ratio range (from 1:9 to 9:1), the CD study showed that the content of *ß*-sheet structure increased as compared to those of monocomponent peptides (Figure 4B), which means the mixtures were easier to self-assemble. We propose that the changes of the secondary structures in the mixtures of the two peptides could be a cause for the change of the morphology of the assemblies.

### MD Simulations

**FIGURE 5:**
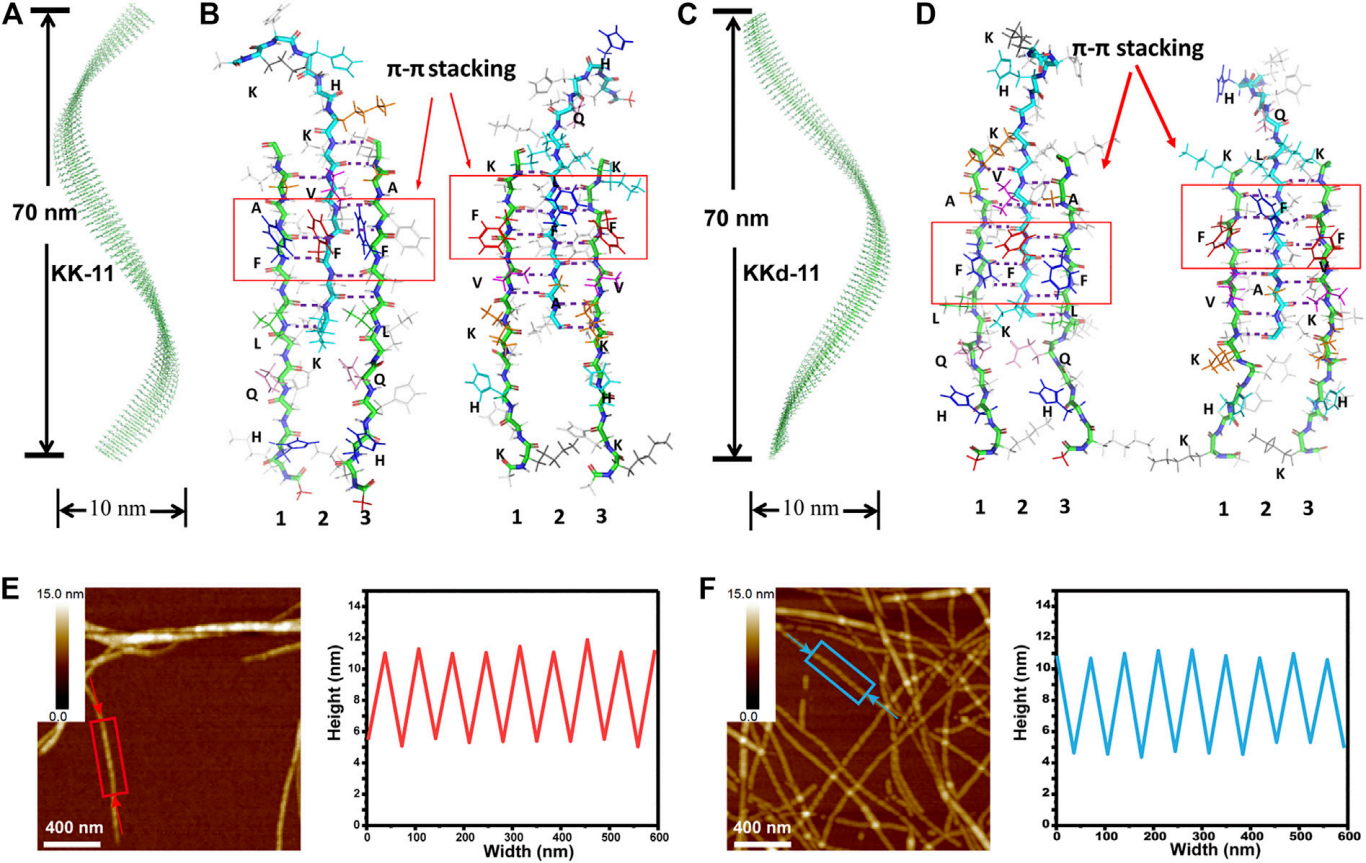
**(A)** The constructed right-handed helical KK-11 fibril with a pitch of 70 nm and a width of 10 nm. **(B)** Inter-stand packing modes of three KK-11 molecules. The hydrogen bonds formed between the hydrophobic region (LVFFA) are shown and the π-π stacking is highlighted with red rectangles. The right three peptides were obtained by rotating the left three peptides 180° to illustrate both sides of the amino acids’ sidechain clearly, with the side that opposite to the reader colored by light grey. **(C)** Left-handed helical KKd-11 fibril with a pitch of 70 nm and a width of 10 nm. **(D)** Inter-stand packing modes of three KKd-11 molecules with similar hydrogen bonding and π-π stacking as (B). The left and right panels were obtained with the same procedure as (B). **(E)** AFM image (left) of fibrils self-assembled with KK-11 and linear section analysis (right) of the peptide fibril highlighted with a rectangle in the AFM image; (F) AFM image (left) of fibrils self-assembled with KKd-11 and linear section analysis (right) of the peptide fibril highlighted with a rectangle in the AFM image. The arrows in (E) and **(F)** indicate the starting and ending points of the section analysis lines.

For monocomponent peptides KK-11 and KKd-11, to understand the molecular basis of the macrochirality of the self-assembled peptide nanofibrils, a series of MD simulations were carried out. According to the above spectroscopic results, the peptides were assumed to take *ß*-sheet conformations. Several different kinds of assemblies of KK-11 and KKd-11 were constructed and were then simulated to test their stability. It was found that the KK-11 and KKd-11self-assembled into stable single-layered helical fibrils (Figures 5A–D) rather than bilayered helical fibrils. The fibrils were either right-handed (KK-11, Figure 5A) or left-handed (KKd-11, Figure 5C) with a pitch of ∼70 nm and a width of ∼10 nm, which are in good agreement with the AFM results (Figures 5E,F).

Peptides KK-11 and KKd-11 could be roughly regarded as the combination of a hydrophilic part (KHHQK) and a hydrophobic part (LVFFA). The hydrophobic part was energetically unfavorable to be solvated, and the hydrophobic interaction acted as the main driving force for the aggregation of the peptides. In the fibrils, the peptides assembled into *ß*-sheet, and hydrogen bonds were formed between adjacent peptides at the hydrophobic part (Figures 5B,D). At the same time, the hydrophilic part of the peptides extended to the solvent. Interestingly, the hydrophobic part did not form bilayered structures with another *ß*-sheet peptide such that the hydrophobic amino acids could be buried inside the fibril. After careful examination of the assembled structures, it was found that the π-π interaction between sidechain of Phes (F) could partly reduce the water-accessible area of the assembled *ß*-sheet. The intermolecular hydrogen bonds were not maximally formed as that of other fibrils reported previously (Swanekamp et al., 2012b; Wang et al., 2017). There may be two reasons for such a construction pattern. First, the hydrophilic amino acids could still form hydrogen bonds with water molecules when not forming hydrogen bonds with adjacent peptides, which was entropic more favorable. Second, the π-π interaction between Phes also enhanced the stability of this assembled fibril. As shown in Figures 5B,D, there are five and six hydrogen bonds between peptides 1, 2, and peptides 2, 3, respectively. In this way, the *ß*-sheets formed by KK-11 and KKd-11 are not symmetrical and exhibited a strong propensity to twist in right-handed and left-handed directions, correspondingly.

**FIGURE 6:**
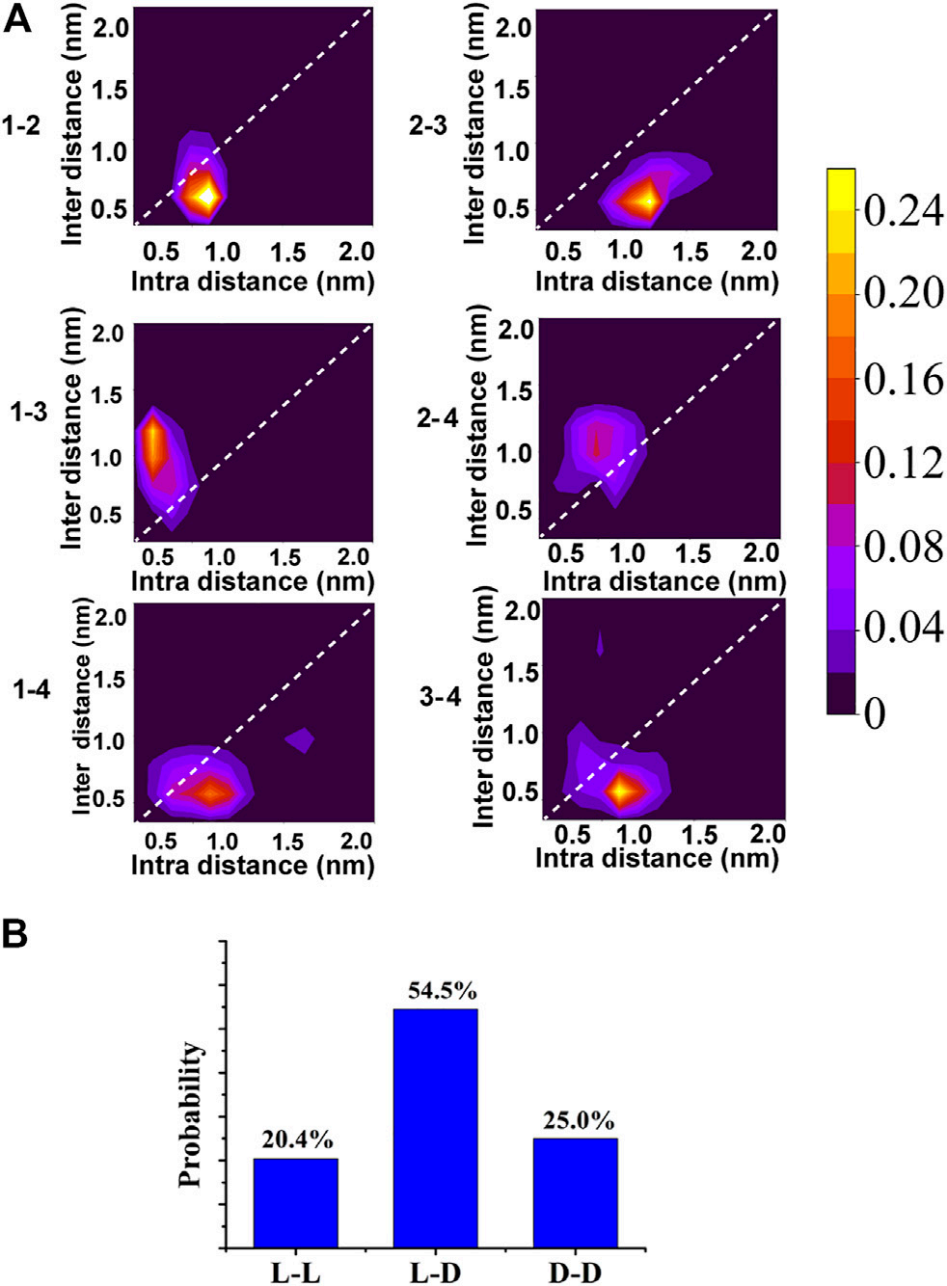
**(A)** The probability map of structures sampled for every two monomers in one of the five simulated mixed systems. Samples are projected into intra-distance (distance between the two monomers) and inter-distance (the nearest distance between the other monomers and either of the two monomers). The probability value corresponding to each color was shown in the color bar. **(B)** The normalized probabilities of the nearest distance attributing to L-L, L-D, D-D.

When KK-11 and KKd-11 were mixed, neither left-handed nor right-handed structure was formed, indicating that there were interactions between peptides KK-11 and KKd-11. A series of MD simulations were then carried out to investigate this phenomenon. Five systems that each contained two KK-11 (e.g., peptide one and two in Figure 6A) and two KKd-11 (e.g., peptide three and four in Figure 6A) of different initial conformations were simulated for 1 µs Interestingly, it was found that two peptides with opposite chirality preferred to interact with each other. In Figure 6A, the probability maps of structures sampled for every two monomers in one of the five simulated systems are shown. Sample on the super diagonals means these two monomers are in the closest contact, whereas sample on the sub diagonals means other monomers are in closer contact with either one of these two monomers. Among all the probability maps of two monomers, only peptides one to three and peptides two to four have high-density areas in the super diagonals, indicating that peptides one to three and peptides two to four are contacted with each other most closely.

To make it clearer, for every two peptides, we counted the occurring number of the most closely contact (Figure 6B). It was found that, in 54.5% of the samples, peptide KK-11 and peptide KKd-11 had the nearest distance, indicating that peptides do have a preference to interact with a different chiral type of peptide.

**FIGURE 7:**
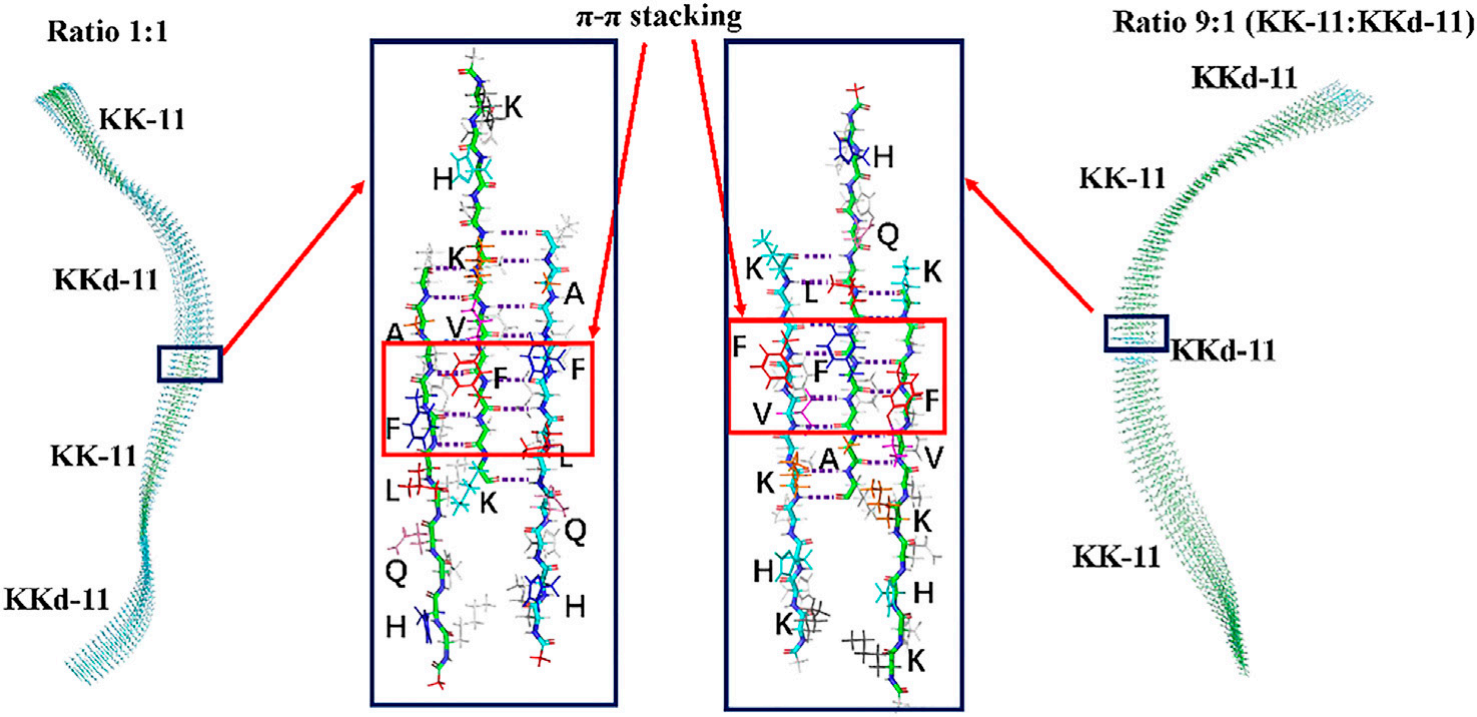
The co-assembled structures were composed of short helical fragments of KK-11 and KKd-11. The junction structures are enlarged in the blue rectangles, in which the hydrogen bonds formed between the hydrophobic region (LVFFA) are shown and the π-π stacking is highlighted with red rectangles. **(A)** The sidechains of amino acids protruding to the reader; **(B)** The sidechains of amino acids protruding to the back. The right three peptides were obtained by rotating the left three peptides 180° to illustrate both sides of the amino acids’ sidechain clearly, with the side that opposite to the reader colored by light grey.

The co-assembled structures were designed by split the helical structures into small segments. As shown in Figure 7, the co-assembled structures of KK-11 and KKd-11 were composed of short helical fragments of either KK-11 or KKd-11. Generally, different ratios of KK-11 and KKd-11 led to different lengths of the short helical fragments, with no intrinsic difference in nature. Previous simulations showed that peptide has a preference to interact with the peptide of a different chirality; however, it is still not likely to form assembled structures in which L-type and D-type peptides are alternatively arranged in a one-by-one fashion, which is entropically unfavorable. Though L-type and D-type peptides have a preference to co-assemble, they can also self-sort, and once formed they would not likely dissociate. For the junction part, there were more hydrogen bonds between KK-11 and KKd-11 molecules than that in monocomponent peptide systems (6 vs. 5.5 hydrogen bonds per peptide). Therefore, it is a little energetically favorable for the peptide to interact with the peptide with different chirality, which is consistent with the statistical results in Figure 6. Experimentally, there were no helical structures observed in the mixed KK-11/KKd-11 system (Figures 2, 3), even at the very early stage of the co-assembly process. Combining these results, we propose that the shorter helical structures were unstable and would soon relax to plain fibril structures. As for the pure KK-11 or KKd-11 systems, the helical structure was long enough to endure thermal fluctuation and would be stable for a much longer time.

These results provide explanations for the formation of the non-helical fibers (Figures 2, 3) in the mixed systems. Even when very few KKd-11 (or KK-11) peptides were mixed with the KK-11 (or KKd-11) peptides, they could participate in the very early stage of assembling and formed stable structures with KK-11 (or KKd-11). In this way, the KKd-11 (or KK-11) peptides split the original helical structure into small segments, which were only metastable structures and will soon relax to plain fiber structures. As a result, achiral nanofibers were formed in the mixed systems even when the concentrations of the two peptides were remarkably different.

## Conclusion

In conclusion, the self-assembled morphologies of two chiral amyloid peptides, KK-11 and KKd-11, with the same amino acid sequence but different types of chirality of their amino acid residues were studied. Topological characterizations by AFM and TEM revealed opposite chiral helixes were formed by KK-11 and KKd-11, respectively, while in the mixtures of the two peptides only achiral nanowires were formed in a wide range of peptide concentration ratios. MD simulations indicated that KK-11 or KKd-11 exhibited a strong propensity to form right-handed or left-handed nanofibrils, respectively. However, when KK-11 and KKd-11 were both presented in a solution, they had a higher probability to interact with each other instead of self-interaction. By considering the entropic issue, we have constructed the co-assembled structures of KK-11 and KKd-11, in which the helixes of a self-sorted peptide were split into small segments by the other peptide so that the local chiral structures were relaxed to achiral fiber structures. The morphology and handedness features of the self-assembled and co-assembled nanostructures are in good agreement with experimental observations. Our study shed light on the molecular mechanisms of the macrochirality of peptides’ supramolecular structures. We believe that this study can help to better understand the origin of chiral in biosystems and to explore the applications of complementary-chirality designs at the molecular and supramolecular levels.

## Data Availability

The datasets presented in this study can be found in online repositories. The names of the repository/repositories and accession number(s) can be found in the article/Supplementary Material.
